# Naturally disengaging control to reveal habits

**DOI:** 10.21203/rs.3.rs-5773028/v1

**Published:** 2025-01-20

**Authors:** Sarah Oh, Anne G.E. Collins

**Affiliations:** Department of Psychology, University of California, Berkeley

## Abstract

Habits are an essential part of everyday decision-making. However, the mechanisms underlying habit formation and expression in humans are difficult to study in the laboratory, owing to a dearth of convenient experimental paradigms that reliably exhibit a key marker of habits – training-induced inflexibility – under ecologically valid conditions. This difficulty is often attributed to the fact that habits are identified in contrast to goal-directed (GD) control, which research participants typically engage strongly in laboratory experiments. To address this gap, we develop a new, short habit learning paradigm that incorporates several features we hypothesized would encourage participants to disengage GD control, enabling habits to exert greater influence over behavior: a hierarchical multi-step trial structure, opportunities for self-correction, and frequent switches between extensively and moderately practiced behaviors. Through a series of experiments, we demonstrate that overtraining amplifies habitual control, as evidenced by errors biased toward the overtrained context and away from the moderately-trained context at early response times, while later responses remain dominated by GD control. The reliability of this overtraining effect depended on the inclusion of task features designed to dampen GD control. In addition to providing a practical, robust, and flexible tool for studying the cognitive processes underlying habit formation and habitual control, our paradigm moves us beyond the traditional stimulus-response conception of habits, expanding the definition to include more complex, hierarchical behaviors that better reflect naturalistic human habits.

## Introduction

A habit is a stable pattern of behavior that has become routine and effortless, even automatic and difficult to change, through frequent repetition. Habits are hugely consequential; a considerable portion of our day-to-day behaviors are habitual (Wood et al., 2002), and when adaptive, habitual control can be effective and efficient, limiting the need for costly cognitive control and freeing up cognitive resources. Conversely, because they are highly automatized, habits can also be associated with behavioral inflexibility and maladaptive decisions, and are thought to play a role in multiple psychiatric disorders, such as obsessive-compulsive disorder (OCD) (Gillan et al., 2011), addiction (Everitt and Robbins, 2016), and anorexia nervosa (Uniacke et al., 2018).

Habits become evident in scenarios where they conflict with one’s current goals but are carried out nonetheless due to automatization. As such, habits are typically characterized in contrast to goal-directed (GD) behavior. Studies using outcome devaluation and related paradigms in rodents have shown that a behavior that, early in learning, is directed toward achieving a particular outcome, gradually comes to be performed automatically, even when the environment changes such that the behavior no longer reliably results in the desired outcome (Adams, 1982; Dickinson et al., 1995; Killcross and Coutureau, 2003). These paradigms have been combined with lesion studies to demonstrate that habitual and GD processes rely on partly dissociable neural networks: GD learning can be disrupted while leaving habit formation intact by damaging certain brain areas such as the dorsomedial striatum, and vice versa by damaging other brain areas such as the dorsolateral striatum (Killcross and Coutureau, 2003; Balleine and Dickinson, 1998; Yin et al., 2004, 2005; Lingawi et al., 2016).

The success of these classic approaches in explicating different forms of behavioral control and their neural mechanisms in non-human animals has inspired efforts to translate them to humans. These have met with mixed success (Tricomi et al., 2009;de Wit et al., 2018; Pool et al., 2022; Gera et al., 2023), suggesting that existing experimental designs may be inadequate for reliably inducing and/or revealing habits in humans. Indeed, behavior in many tasks developed to study habits in humans does not exhibit the key marker of habits: sensitivity to overtraining (de Wit et al., 2018, 2013). Furthermore, behavior on these tasks tends to correlate with individual measures related to GD control, rather than with other behavioral or self-report measures purported to relate to real-life habits (Gillan et al., 2015; Friedel et al., 2014; Sjoerds et al., 2016; Linnebank et al., 2018; Nebe et al., 2024). Together, these results suggest that existing task paradigms are more sensitive to individual differences in GD control processes than to individual differences in processes supporting habit formation or habitual control.

Successful demonstrations of overtraining-induced habits in humans often rely on additional experimental constraints, such as acute stress (Schwabe and Wolf, 2009) or forced response times (Hardwick et al., 2019), that do not reflect the conditions under which habits emerge in real life settings, but work to decrease the amount of GD control participants can exert. Consequently, their results hint that the difficulty in demonstrating habits in human laboratory tasks may lie in inducing participants to release cognitive control in laboratory experiment settings (Buabang et al., 2024). To address this, we developed a new paradigm that extends Hardwick et al. (2019), incorporating several features designed to naturally encourage participants to disengage GD control, allowing habits to exert control over behavior.

First, we embedded choices into a hierarchical multi-step trial structure, spreading out each trial over three dependent decision-making steps. We drew inspiration from research on chunked action sequences, which share both behavioral features – such as increased speed, consistency, and inflexibility with training (Balleine et al., 1995; Ostlund et al., 2009; Dezfouli et al., 2014) – and neural substrates (Jin and Costa, 2010; Thorn et al., 2010; Smith and Graybiel, 2013) with habits. This design aims to 1) tax GD control by requiring participants to maintain and monitor a plan across steps, and 2) facilitate the emergence of habitual behavior by encouraging deliberate selection of abstract plans, followed by more automated execution at later steps. We hypothesized that increasing the complexity of trained behaviors beyond single stimulus-response (S–R) associations would make it less trivial for human participants to override habitual impulses with GD control.

Second, we included both extensively practiced and minimally practiced contexts when testing for habits, forcing participants to switch between a putatively habitized behavior and a behavior requiring GD control. Previous research has shown evidence that this may help in revealing habits (Luque et al., 2020). We hypothesized that requiring participants to flexibly switch between overtrained and moderately-trained contexts would 1) challenge the GD system, allowing habitual control to emerge more readily, and 2) provide a more sensitive measure of habit strength by combining evidence of inflexibility in novel contexts with evidence of fluency in familiar contexts.

Third, we allowed participants to self-correct their responses, motivated by the insight that habitual and goal-directed control are partly separate processes that follow distinct temporal dynamics. Hardwick et al. (2019), who following a contingency reversal forced participants to respond to stimuli at controlled time delays, found that overtrained participants exhibited more slips of action toward the original S–R mapping when forced to respond after short 300–600 ms delays, but not after longer delays. We reasoned that allowing multiple opportunities to respond would 1) reduce participants’ response inhibition, allowing us to observe slips of action in their early responses without artificially constraining their response times, and 2) enable us to assess habit strength separately from the strength of GD control which would dominate choices at later response times.

Building on Hardwick et al. (2019), we operationalized habits based on a contingency reversal, using slips of action to characterize behavioral inflexibility, and extended the protocol to include a hierarchical trial structure, both pre- and post-reversal contexts in the test phase, and the allowance for multiple responses to test phase trials. Using our novel paradigm, we successfully detected an effect of overtraining such that extended practice amplified habitual control, as evidenced by decreases in slips of action in the pre-reversal context and increases in slips of action in the post-reversal context; further, we verified that the task features we added contributed to this effect. Importantly, we revealed in-lab habit formation within a few hundred iterations with a task duration of less than one hour, making this paradigm both practical and in line with the dynamics of real-life habit formation (Buyalskaya et al., 2023; Lally et al., 2010). Our experiment disengages cognitive control naturally, without relying on artificial constraints, allowing the habitual system to exert control over behavior in situations that more closely reflect real-life choice conditions than previous paradigms, providing a valuable tool for advancing our understanding of the mechanisms underlying habit formation and expression.

## Results

We administered seven versions (Experiments 1a, 1b, 2a, 2b, 2c, 2d, and 3) of a task, with total task duration ranging from approximately 30 to 60 minutes depending on task version and training group. Task variants were developed to test the importance of various task design choices on our protocol’s ability to reveal habit formation. Table 1 summarizes the features of each task version (see Methods for details of task implementation, data collection, and exclusion criteria; see Supplementary Information for a link to a short demo of Exp 1b).

**Figure 1:**
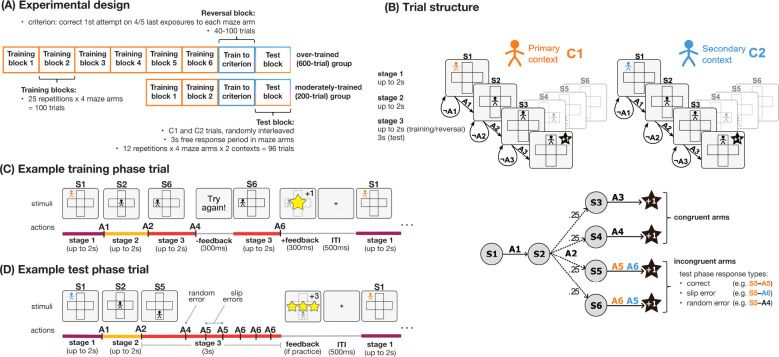
Task design for Experiment 1a. The features depicted in this figure are accurate for most of the other experiments, but see Methods for a detailed description of the features unique to each task version. **(A)** Number of trials in each phase of the task, for the overtrained and moderately-trained groups. **(B)** Structure of each trial. Stage 1 of each trial began in the starting state (S1), from which selecting the correct key press action led to stage 2 of the trial (S2). Choosing correctly from S2 led to stage 3, which presented a random terminal arm of the maze (S3-S6) with uniform probability. Choosing the correct key press from the maze arm led to reward (a star, worth 1 point). Selecting the incorrect key press action or failing to respond within the allotted time at any stage led back to the same state after flashing an instruction message (except stage 3 of test phase trials, when no feedback was shown for correct or incorrect responses). Context information (avatar color) was only available in stage 1 of each trial. Two of the four maze arms had their correct key presses swapped from Context C1 to C2, similar to Hardwick et al. (2019); we refer to these as incongruent arms, and the two arms with consistent stimulus-action mappings across contexts as congruent arms. Responses to incongruent arms during the test phase can either be the correct response, a slip error (correct response for opposite context), or a random error (any other error). **(C)** An example sequence of stimuli and actions that a participant might experience during the training phase. The participant begins in S1, selects the correct action A1 and is presented with S2, selects the correct action A2 and is presented with a random maze arm, in this case S6. The participant selects an incorrect action and is shown feedback, then presented with S6 again. The participant selects the correct action A6 and receives reward feedback, followed by an inter-trial-interval (ITI), after which the next trial begins with the presentation of S1. **(D)** An example sequence of stimuli and actions that a participant might experience during the test phase. The participant is presented with S1 in Context C2, selects the correct action A1 and is presented with S2, selects the correct action A2 and is presented with S5. The participant makes several responses during the 3-second stage 3 response period, without feedback (except in practice trials), followed by an ITI and the start of the next trial, this time in C1.

All experiments shared the same core task design (Exp 1a, Fig. 1) aimed at testing slips of action when overtrained, similar to Hardwick et al. (2019), with additional features based on mechanisms we hypothesized would naturally encourage participants to disengage control. Participants were first introduced to the task environment’s primary context (C1), indicated by an orange avatar. In C1, six avatar positions on a simple cross-shaped maze (S1-S6, corresponding to the outside the of maze, the center of the maze, and the top, right, bottom, and left arms of the maze) were each associated with a key press action (A1-A6, each corresponding to one of S, D, F, J, K, and L); participants learned these associations by trial and error. Each trial proceeded in three stages (trial structure illustrated in Fig. 1B; example training phase trial shown in Fig. 1C). In stage 1 of each training phase trial, the orange avatar was presented in S1 (outside the maze); pressing A1, the correct key for S1, moved the avatar to S2 (maze center), beginning stage 2. In stage 2, pressing A2 (the correct key for S2) moved the avatar to a random arm of the maze (e.g. S6, the left maze arm), with .25 probability assigned to each of the four maze arms (S3-S6). In stage 3, pressing the correct key for the current maze arm (e.g. A6 for S6) resulted in positive feedback and ended the trial. The presence of three stages enabled us to manipulate the hierarchical embedding of the stimulus-action associations learned, a feature we hypothesized would support diminished control.

Following either 200 or 600 training phase trials, depending on the participant’s assigned experimental group (moderately-trained or overtrained), participants entered the reversal phase, which continued until they reached a training criterion (correct first attempt on four out of the five most recent exposures to each maze arm) after completing a minimum of 40 trials, or completed 100 trials, whichever came first. The reversal phase presented trials in a secondary context (C2), indicated by a blue avatar, wherein the stimulus-response (S–R) associations for two of the maze arms (incongruent arms) were reversed with respect to C1 (e.g. S5-A5 and S6-A6 became S5-A6 and S6-A5, Fig. 1B), while the S–R associations remained unchanged for the other two maze arms (congruent arms). This approach of reversing contingencies for a subset of stimuli while leaving others unchanged was inspired by previous work that used a similar design to probe the dynamics of habitual and goal-directed control (Hardwick et al., 2019). Unlike the Hardwick et al. (2019) task which only tested participants on the post-reversal contingencies with forced response times, the test phase in our design included both C1 and C2 trials, randomly interleaved, and participants were allowed to make multiple responses in stage 3 of each test phase trial; again, this was aimed at encouraging participants to release control. They received no feedback but were instructed that they would collect one point for each correct response (example test phase trial shown in Fig. 1D). Throughout the task, the avatar appeared in black during stages 2 and 3, so context information was only available in stage 1 of each trial, forcing participants to hold the current context in mind while executing stages 2 and 3.

To evaluate whether participants developed habitual choices, we analyzed the types of errors participants made when responding to incongruent maze arms during the test phase; following Hardwick et al. (2019), we call an erroneous response a “slip error” if it corresponds to the correct response for the opposite context, and a “random error” otherwise (Fig. 1B,D). Because C1 was practiced more extensively than C2, we expected the behavior learned in C1 to be more ingrained than that learned in C2, and that this would emerge as a higher rate of C1-consistent slip errors in C2, and a lower rate of C2-consistent slip errors in C1. We thus defined a behavioral Habit Index (HI) as follows:

(1) $$HI=\left(P_{\mathrm{slip}}^{C2}-P_{\mathrm{rand}}^{C2}/2\right)-\left(P_{\mathrm{slip}}^{C1}-P_{\mathrm{rand}}^{C1}/2\right)$$

This HI controls for slip errors that might be driven by other factors that vary with overtraining (such as practice or fatigue effects) but are not due to overtraining itself, allowing us to attribute differences in baseline-corrected slip error rates specifically to the effect of overtraining (see Methods). If the Habit Index is more positive in overtrained participants than in moderately-trained participants, it would provide evidence that our task can induce habits that are strengthened as a function of training duration.

Experiments 1a and 1b had an identical structure, with only minimal differences aimed at improving exclusion rate. Here, we focus mainly on presenting behavior and analyses for Exp 1b, with N=44 in the 200-trial group and N=48 in the 600-trial group (see Fig. 5, Fig. S1 and Table S2 for Exp 1a and all other experiments).

### Participants’ test phase errors reflect deviations from goal-directed control.

**Figure 2:**
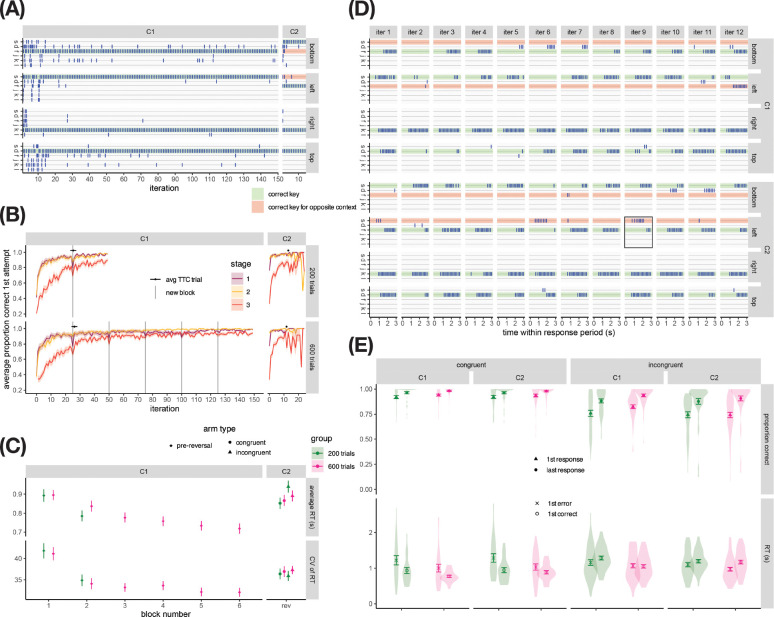
**(A)** All stage 3 responses from a representative participant’s training and reversal phase behavior. Rows corresponding to the correct key press response are highlighted in green, while rows corresponding to the slip error response are highlighted in red. **(B)** Average learning curves across participants (ribbons indicate mean ± s.e.m.). Vertical gray lines indicate block boundaries. The horizontal black lines show the mean ± s.e.m. number of trials to criterion (TTC) across participants. Large variance in iterations 10–20 of C2 reflect fewer participants’ data points as C2 was trained to criterion, with a minimum of 10 iterations. **(C)** Average RT (top) and coefficient of variation of RT (bottom) across task blocks, by trial type (error bars indicate mean ± s.e.m. across participants). **(D)** All stage 3 responses from the same representative participant’s test phase behavior. A trial in which the participant initially makes habit-consistent slip error responses, then switches to the correct response, is highlighted. **(E)** Top, distribution across participants of the accuracies of participants’ first and last responses by congruent/incongruent arm type and context, computed by averaging across test phase trials (error bars indicate mean ± s.e.m. across participants). Bottom, distribution across participants of the RTs of participants’ first incorrect and the first correct responses, by congruent/incongruent arm type and context (excluding 46 participants who made either 0 incorrect or 0 correct responses to congruent arms in either C1 or C2 for the congruent arm panel, left, and similarly excluding 10 participants for the incongruent arm panel, right; error bars indicate mean ± s.e.m.).

We first verified that participants were able to learn the associations well in the first training phase in both groups (see 2A for an example participant, and Fig. 2B for an aggregate). Indeed, the accuracy of participants’ choices at each stage improved both across and within training phase blocks in both groups; accuracy dipped at the beginning of the reversal phase, then increased quickly as participants learned the new S-R mappings for incongruent arms (Fig. 2B). On average, the moderately-trained group took 52 (SD=16.3) trials to reach criterion (TTC) in the reversal phase, while the overtrained group took 47.1 (SD=14.3) TTC; there was no significant effect of group on TTC ($F(1,\;90)=2.43,\;p=.123,\;\eta_{G}^{2}=0.026$). If we were to evaluate a similar criterion for the training phase, the moderately-trained group would take 101 (SD=53.1) trials, and the overtrained group would take 105 (SD=55.9), with no significant effect of group ($F(1,90)=0.084,\;p=.773,\;\eta_{G}^{2}=0.001$). During the training phase (C1), both groups showed a progressive decrease in RT and RT variability across blocks as participants became more familiar with the task contingencies (Fig. 2C), a standard marker of choice automatization (Jin and Costa, 2010; Du et al., 2022). RT and RT variability increased when the task contingencies were altered in the reversal phase (C2). A two-way mixed ANOVA testing the effect of group and congruent/incongruent arm type on average RT in C2 revealed a significant main effect of arm type ($F(1,90)=15.1,\;p<.001,\;\eta_{G}^{2}=0.019$), due to participants responding more slowly on incongruent trials. While there was no significant main effect of group, ($F(1,90)=0.188,\;p=.666,\;\eta_{G}^{2}=0.002$), there was a significant interaction between group and congruent/incongruent arm type, ($F(1,90)=4.79,\;p=.031,\;\eta_{G}^{2}=0.006$), reflecting the more pronounced effect of incongruency on response slowing in the moderately-trained group compared to the overtrained group.

Before assessing habit strength using the Habit Index, we aimed to verify that participants in both groups learned the task well and could effectively express goal-directed behavior during the test phase, ensuring that both groups’ errors reflect deviations from goal-directed control, whether due to habitual control or attentional lapses, rather than group differences in task understanding, mastery, or fatigue. Figure 2D displays all of the test phase stage 3 responses from the same participant whose training phase behavior is shown in Fig. 2A; Figure 2E displays the distributions of participants’ accuracies (top) and RTs (bottom), broken down by arm type (congruent/incongruent) and context (C1/C2). We conducted a three-way mixed ANOVA test for effects of group (between-participant), C1/C2 context, congruent/incongruent arm type, and first/last position (within-participant) on the average response accuracy of participants’ first and last responses in each 3s response window. This revealed a significant main effect of position in sequence ($F(1,90)=192.82,\;p<.001,\;\eta_{G}^{2}=0.107$), driven by an increase in accuracy from participants’ first to their last responses, indicating that participants corrected early errors. We also observed a significant main effect of arm type, ($F(1,90)=109.36,\;p<.001,\;\eta_{G}^{2}=0.173$), driven by more accurate responses in the congruent vs. incongruent maze arms; and a significant interaction between position in sequence and arm type, $F(1,90)=72.72,\;p<.001,\;\eta_{G}^{2}=0.031$, driven by a larger increase in accuracy from first to last responses in the incongruent relative to the congruent arm condition (Fig. 2E). Importantly, there was no significant main effect of group, nor significant interactions involving group, indicating that overtrained participants and moderately-trained participants had similar accuracies across first/last responses, congruent/incongruent arms, and C1/C2 contexts (all F<2.25,p>.137). Thus, both groups acquired good knowledge of task contingencies during the training and reversal phases and were able to use their knowledge effectively during the test phase, allowing us to interpret the types of errors participants made (e.g., slip errors) as deviations from goal-directed control rather than as artifacts of poor learning.

Next, we explored the temporal dynamics of participants’ responses. We expected that first errors would be faster than first correct responses in incongruent arms, reflecting impulses from the competing context, and that overtraining would amplify this effect in C2 and dampen it in C1. A two-way mixed ANOVA tested for effects of group, context, and response type (first error / first correct) on the average RTs of participants’ first incorrect and first correct responses across incongruent trials (participants with 0 errors in any combination of conditions were excluded from this analysis, leaving 82 participants’ data). There was a significant main effect of response type $F(1,80)=14.77,\;p<.001,\;\eta_{G}^{2}=0.019$, reflecting *shorter* RTs for first errors vs. first correct responses (Fig. 2E), as expected; there was no significant main effect of group, but there was a significant three-way interaction between group, context, and response type $F(1,80)=5.12,\;p=.026,\;\eta_{G}^{2}=0.007$, indicating that while first errors are generally faster than last errors in incongruent arms, the magnitude or even direction of this difference in RTs depended on the combination of group and context. Post-hoc tests revealed in the 200-trial group a significant main effect of response type ($F(1,36)=6.46,\;p=.015,\;\eta_{G}^{2}=0.022$), but no significant interaction between response type and context, indicating that the tendency to make fast errors was similar across contexts; pairwise t-tests showed that first errors were on average faster than first correct responses in both contexts, significantly so in C1 (t(36)=−2.10,p=.042) but not C2 (t(36)=−1.41,p=.168). In the 600-trial group, there was again a significant main effect of response type ($F(1,44)=8.41,\;p=.006,\;\eta_{G}^{2}=0.017$), and additionally a significant interaction between response type and context, indicating that the tendency to make fast errors differed across contexts; pairwise t-tests indicated that first errors were significantly faster than first correct responses in C2 (t(45)=−4.05,p<.001) but were on average slower than first correct responses in C1 (though the difference was not significant t(45)=0.41,p=.688). Thus, while moderately-trained participants showed similar speeding of errors with respect to correct responses in C1 vs. C2, overtraining led participants to make relatively slower errors in C1 and relatively faster errors in C2 – consistent with our high-level prediction that overtraining would exacerbate impulsive responding in C2, but not C1.

A separate two-way mixed ANOVA for congruent arms (46 participants included in analysis) revealed a significant main effect of response type ($F(1,44)=17.03,\;p<.001,\;\eta_{G}^{2}=0.084$), reflecting longer RTs for first errors vs. first correct responses; and a significant main effect of group $F(1,44)=4.74,\;p=.035,\;\eta_{G}^{2}=0.042$, reflecting an increase in fluency of responding with practice (Fig. 2E). While first errors in incongruent arms may be driven by quick impulses, the relative speed of first correct responses vs. first errors in congruent arms suggest that first errors in congruent arms may be due to confusion.

Taken together, these results suggest that both moderately-trained and overtrained participants were able to exercise goal-directed control in test phase choices, and that any deviations from goal-directed control owing to automatic habitual impulses would be most evident in incongruent arms, and in early responses.

### Overtraining increases the Habit Index.

**Figure 3:**
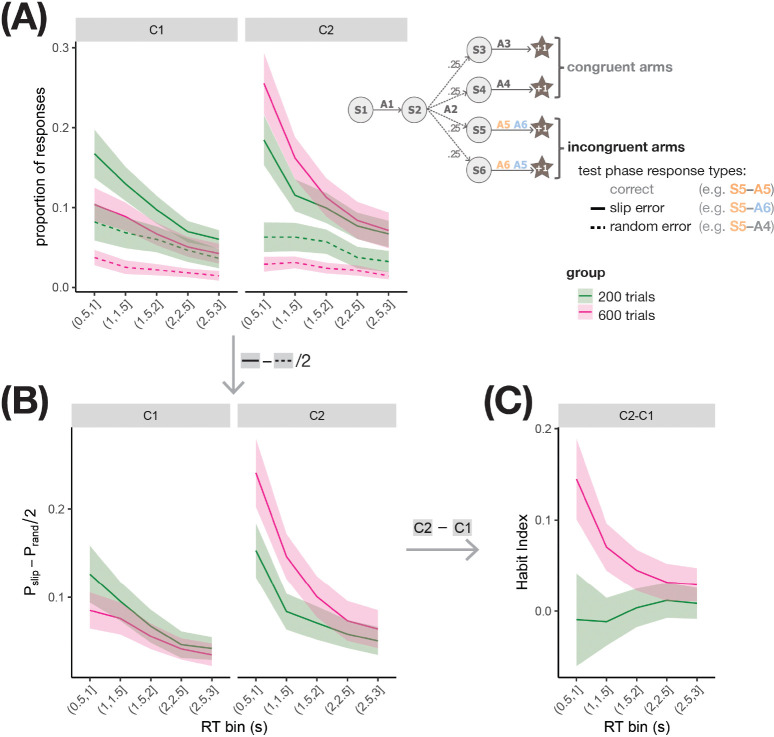
Computation of Habit Index. Lines and ribbons indicate mean ± s.e.m. across participants. **(A)** Proportion of responses that are slip errors (solid) and random errors (dashed) for each combination of group (200-trial, pink; and 600-trial, green), context (C1, left; and C2, right), and RT (0.5s bins spanning the 3s response window). **(B)** The baseline-corrected slip error rate (P_slip_ − P_rand_/2) by group, context, and RT. **(C)** The strength of the tendency to have a higher baseline-corrected slip error rate in C2 vs. C1, i.e. the Habit Index, by group and RT.

Reasoning that any effect of training duration on habitual control should be most discernible in early responses to incongruent arms, we next focused on analyzing the types of errors participants made in incongruent arms, summarized as the Habit Index (HI, see Methods), in time bins spanning the 3s response period. If we successfully induced habits in the overtrained, but not the moderately-trained group, we should expect to see a stronger HI in the overtrained group, especially early in the trial; later in the trial, participants might exert control to correct their habitual responses, leading to a weak HI in both groups. Indeed, as shown in Figure 3, in early time bins (~0.5–1.5s), overtrained participants on average exhibited larger values of HI relative to moderately-trained participants; the difference between training groups largely disappeared in later time bins (~2–3s).

Because the Habit Index and the quantities used to compute it were not normally distributed (see Fig. S2 for plots showing individual data points), we performed a bootstrapping analysis to generate confidence intervals based on the empirical distribution of our data, in order to assess how confident we should be in the overtraining effect (see Methods for details). Table 2 displays for each summary measure (slip error rate, random error rate, and baseline-corrected slip error rate) contrasts computed from the measure for two groups, contexts, and/or RT bins, and the proportion of bootstrap samples in which the contrast was greater than zero when sampled participants retain their true group labels. Values of %>0 near 100 indicate that across bootstrap samples, the contrast was reliably positive. For contrasts between groups, the table also displays $p_{\mathrm{null}>emp}$, the proportion of bootstrap samples in which 600-trial minus 200-trial group differences observed in the full dataset are smaller than those computed in the bootstrap samples when the group labels are shuffled to yield a null distribution. $p_{\mathrm{null}>emp}$-values near zero indicate that a difference as large as that observed in the full dataset is unlikely to have occurred by chance.

Consistent with the pattern seen in Figure 3C, HI in the early RT bin is reliably positive in the 600-trial group (99.4% of bootstrap samples with HI>0); the same is not true of the 200-trial group (44.6% of bootstrap samples with HI>0). The effect of overtraining on the Habit Index at early RTs is robust, with the 600-trial group exhibiting a larger bias toward making baseline-corrected errors in C2 vs. C1 (i.e. a larger HI relative to the 200-trial group) in 96.3% of bootstrap samples (p=.040). Overtraining in the early RT bin is associated with an increased baseline-corrected slip error rate in C2 in 91.1% of bootstrap samples and a decreased baseline-corrected slip error rate in C1 in 82.1% of bootstrap samples, but the empirical group difference observed in the full dataset is not statistically significant for either context (p=.089 and p=.201, respectively), suggesting that both contribute to the significant effect of overtraining on HI in the early RT bin.

The overtraining effect is diminished by the late RT bin, with 73.7% of bootstrap samples favoring a larger Habit Index in the 600-trial group (p=.260), which suggests that overtrained participants recruit cognitive control to override their habitual impulses as they continue to respond. In support of this interpretation, the Habit Index computed for the 600-trial group decreases from the early to the late RT bin in 98.0% of bootstrap samples, but only in 46.1% of samples for the 200-trial group. The decrease in Habit Index from the early to the late RT bin is greater in magnitude in the 600-trial group than in the 200-trial group in 91.6% of bootstrap samples (although the empirical group difference does not reach statistical significance relative to the null distribution, p=.072).

In sum, visualizing the Habit Index and its components in discrete time bins revealed that overtrained participants exhibited larger values of HI relative to moderately-trained participants, especially early in the response period, and the robustness of this effect of overtraining on HI at early RTs was supported by bootstrapping analyses. These results indicate that the training procedure in Exp 1b effectively induces inflexible behavioral impulses through extended practice, and that the test phase procedure is well-suited to revealing these impulses at early RTs.

### Regression analysis and probabilistic modeling further supports the existence of an overtraining effect

To examine how training duration interacts with other variables in generating different types of responses on the task without grouping responses into time bins, we used the brms package in R, which interfaces with the Markov Chain Monte-Carlo sampling tool Stan (Gelman et al., 2015), to estimate the posterior distribution of the coefficients of a regression model predicting whether a response is the correct response, a slip error, or a random error, based on various predictors including training duration and its interaction with other task-related variables.

**Figure 4:**
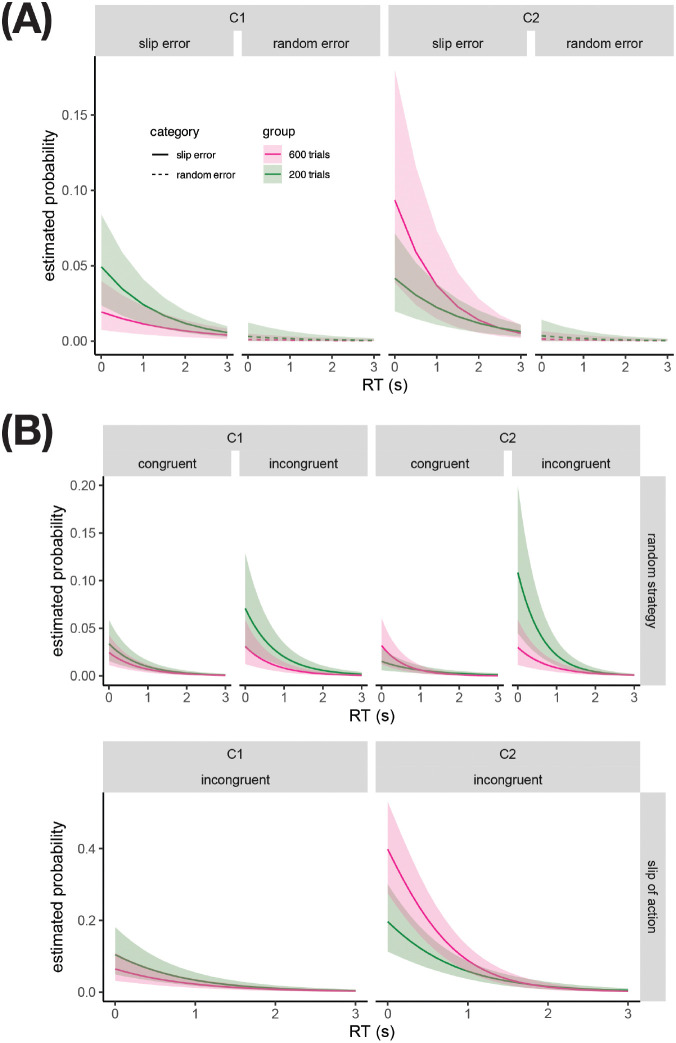
Lines indicate average probability predicted from posterior samples of hierarchical Bayesian model parameters, ribbons indicate 95% CrI. **(A)** brms multinomial regression estimating the probability of slip errors vs. correct responses, and random errors vs. correct responses, as a function of context, group, and RT. **(B)** custom Stan model estimating the probability of deploying a random strategy as a function of context, group, RT, and congruent/incongruent trial type (top), while also estimating the probability of a slip error when on-task (when not under the random strategy) as a function of context, group, and RT (bottom).

After verifying that the predicted probabilities, shown in Figure 4A, qualitatively match the patterns in the slip and random error proportions shown in Figure 3, we used the posterior samples to test hypotheses about how variables influence the probabilities of slip errors, random errors, and baseline-corrected slip error probability. Specifically, we tested whether the extent to which the measures of interest were larger in C2 relative to C1 increased with overtraining, at an early RT (0.5s) and at a late RT (3s). As shown in Table 3, the estimated difference between groups in the difference between C2 and C1 slip error rates was 0.172 (90% CrI [.127, 0.221]) at early RTs. A posterior probability of 1.00 indicated very strong evidence that overtraining increases the C2 minus C1 difference in slip error probability near the beginning of the response period. As expected, the evidence for an effect was weaker at the end of the response period. Similarly, the posterior samples indicated only weak evidence that the difference between C2 and C1 random error probabilities error probabilities is influenced by overtraining, both at early and at late RTs. The estimated difference between groups in the difference between C2 and C1 baseline-corrected slip error rates (i.e., HI, eq. 1) was 0.154 (90% CrI [.109,.204]) at early RTs. A posterior probability of 1.00 indicated that the predicted HI was larger in the 600-trial group than in the 200-trial group for all or virtually all of the posterior samples. Similar evidence is not present at late RTs.

To bypass the assumption that the amount of slip errors driven by random responding is half the random error rate in order to estimate the rate of true slips of action, we fit a custom Stan model that accounts for the fact that slip errors can occur under random responding. The model assumes that response types (correct, slip error, random error) are sampled from a mixture of two distributions: one defined by a random strategy (which can generate any response type), and the other by an on-task strategy (which can only generate slip errors and correct responses). Under the on-task strategy, the probability of slip errors on incongruent trials is a function of RT, context, and training group. The balance between the random and on-task strategies is also estimated a function of RT, context, training group, and congruent/incongruent trial type. That is, the model estimates the probability of responding at random, and the probability of making a slip error when not responding at random (i.e. the probability of a slip of action), as a function of task variables and training group. If the 600-trial group is more biased toward making slips of action in C2 vs. C1 relative to the 200-trial group, it would support the existence of overtraining-induced habits.

We visualized the posterior probabilities, obtained using the posterior samples of fixed effects and intercepts, of responding at random, and those of action slips, for various task conditions for each training group (Figure 4B). The differences between groups are qualitatively consistent with the patterns seen in Figure 3; for example, relative to the 200-trial group, the 600-trial group has higher probability of both slip errors in Figure 3 and slips of action in Figure 4 in Context C2, but the opposite patterns are seen in C1. Formal hypothesis testing on the posterior probabilities showed that an early RT, relative to the 200-trial group, the 600-trial group is more prone to action slips in C2 vs. C1 according to 96% of posterior samples, indicating strong evidence for overtraining-induced habits (Table 4). Similar evidence is not present at the late RT, supporting the idea that the influence of overtrained habits on responding can be suppressed with longer processing times.

### Overtraining effect depends on GD-dampening task features.

**Figure 5:**
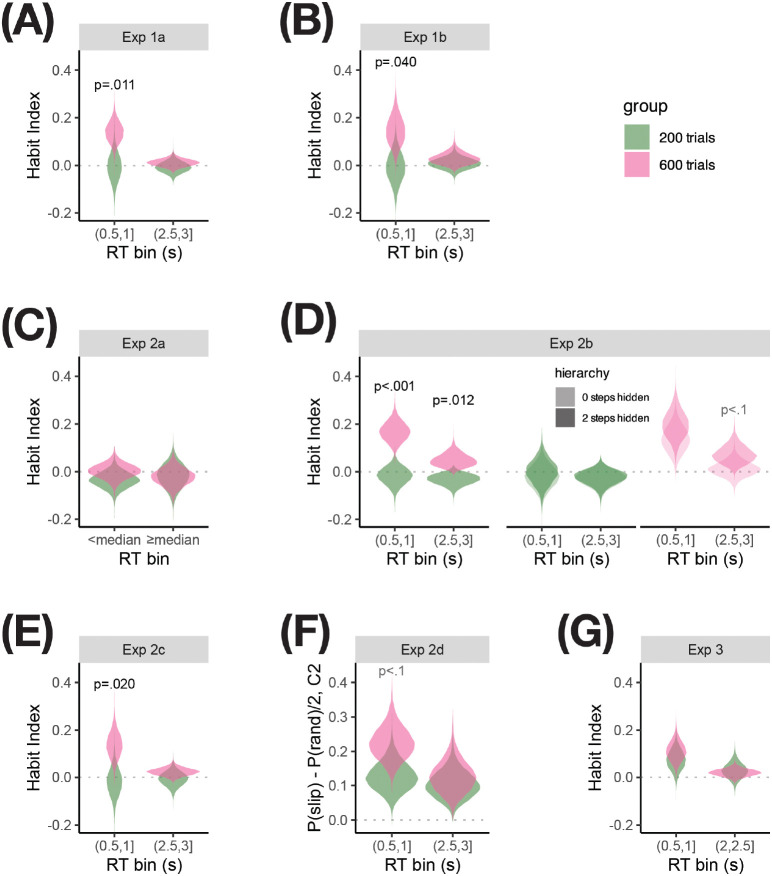
Highlighted bootstrapping results by task version. **(A)** The original version shows identical findings to Fig. 3. **(B)** Gamified version HI (same as figure 3. **(C)** The single-response version shows no evidence of habit. **(D)** Exp 2b manipulated the number of steps over which context information was hidden. The left subplot shows the standard group effect averaged over number of steps hidden, replicating the effect of habit. The middle and right subplots separate the distributions by number of steps hidden, revealing no strong effect of hierarchy. **(E)** Exp 2c has no multi-step structure (with terminal maze arms only), but nevertheless shows weak habit effect. **(F)** C2 only during test. **(G)** within-participant version.

Results from Exp 1b showed that we successfully induced overtraining effects, reflected in a stronger Habit Index (HI) in the 600-trial group compared to the 200-trial group, particularly at early response times. We next sought to test which features of our task were critical to revealing habits in the task. We predicted that features supporting release of control would strengthen our ability to detect overtraining effects. We performed similar bootstrapping analyses as done for Exp 1b for all versions of the task (see Figure 5, and SI).

Experiment 1a was developed first and contained all of the task features we initially hypothesized would encourage habit expression (Table 1). Experiment 1b added task features to Exp 1a that were intended to boost participant engagement, after we found that a substantial number of Exp 1a participants who had ostensibly succeeded in learning the C1 and C2 S–R mappings during the training and reversal phases exhibited signatures of careless or insufficient-effort responding during the test phase (see Methods for details). These task modifications turned out to decrease the reliability with which an overtraining effect was observed (Fig 5a,b). Experiment 1a replicated the main finding from Exp 1b – the HI in the 600-trial group in the early time bin was significantly higher than in the 200-trial group – with stronger quantitative results (empirical group difference of 0.166 with *p* = .011, compared to an empirical difference of 0.154 with *p* = .040 in Exp 1b). The significant but weaker effect in Exp 1b may be due to the increased stakes of responding incorrectly in the gamified task (see Methods for details about task features), which may have encouraged participants to respond with greater engagement of GD control, leading to fewer impulsive responses falling at earlier RTs (see Figure S3 and Table S1). The design of Exp 1a aligns with our overall approach of including task features that reduce GD control to reveal habits. However, a negative side-effect of this approach was that more participants exhibited satisficing strategies, leading to a higher exclusion rate relative to Exp 1b (see Exclusion Criteria).

Exp 2a only allowed one response in stage 3 of each test phase trial, instead of allowing multiple responses; we hypothesized that this would increase the stakes of responding even further than Exp 1b by not allowing participants to self-correct. Although habits were mostly evident in early key-presses of the test trials, we nevertheless expected that the lack of opportunity to correct errors would encourage participants to exercise more deliberate, goal-directed control to inhibit habitual impulses, making it more difficult to detect a habit effect in this version. Indeed, when binning responses by whether they occur before the median participant’s median RT (0.84s) and shuffling group labels, the difference between the 600-trial and 200-trial groups’ average Habit Index in the null distribution was larger than the empirical value in 80.3% of bootstrap samples (*p* = .146), indicating a trend toward an overtraining effect, but not a reliable one (Figure 5c).

Experiment 2b manipulated the availability of context information during the test phase within-participant, making the avatar’s color visible in stage 1 only, in stages 1 and 2, or in all three stages of any given trial, with the number of stages over which the context would be hidden known to the participant from the beginning of the trial. We expected that increasing the degree of hierarchical control needed by requiring participants to hold in mind and execute a more temporally extended abstract plan would allow the decisions at later steps to come under more automatic control, allowing habits to be expressed more strongly at stage 3 when the context was hidden for two steps (similar to Exp 1b) than when context was hidden for one or zero steps. Before testing this, we first verified that the overtraining effect in the early RT bin observed in Exps 1a and 1b was replicated (Figure 5c, left), averaging over all hierarchy conditions (*p* < .001 for effect of overtraining on HI in the early RT bin). Indeed, the effect in Exp 2b was more reliable than in Exps 1a and 1b, which may be due to the larger amount of trials (96 rather than 48 incongruent trials) leading to more stable estimates both within and across participants (see Figure S3 and Table S1). Unlike in Exps 1a and 1b, an overtraining effect was also observed in the late RT bin (*p* = .014); this effect was numerically smaller than in the early RT bin, as expected. When examining the effect of the hierarchy manipulation in the 200-trial group, we found no reliable effect of hierarchy in either the early (*p* = .360) or the late (*p* = .458) RT bin (Figure 5c, middle). In the 600-trial group, however, we found a trend toward an effect of hierarchy in the early RT bin (*p* = .138), suggesting that keeping the context hidden after stage 1 may have weakly contributed to the observation of an overtraining effect in Exps 1a and 1b. We also found a trending effect in the late RT bin (*p* = .077), which may indicate that overtrained participants are less willing to exert the control required to override slips of action when context information needs to be held in mind (Figure 5C, right). We observed a similar trend (*p* = .093, see Table S1) when including only the first 96 test phase trials in the analysis to match the duration of Exps 1a and 1b, suggesting that reduced control at later RTs when context is hidden for 2 steps vs. 0 steps in overtrained participants was not merely due to fatigue.

Experiment 2c eliminated the hierarchical multi-step trial structure altogether, by presenting only stage 3 (the maze arms) throughout the task. Since we expected the multi-stage trial structure to facilitate habit expression in Exp 1a, we expected habits would be more difficult to detect in Exp 2c. Contrary to our expectation, we still observed a reliable overtraining effect (Fig 5D); however, consistent with our hypothesis, the effect appeared to be slightly less reliable than in Exp 1a (*p* = .020, v.s. *p* = .011 in Exp 1a), despite similar numbers of participants with responses in the early RT bin (Figure S3 and Table S1). This suggests that while hierarchy may not be necessary for observing overtraining-induced habits, it may contribute to the reliability of the effect – this would be in keeping with Exp 2b, which found a stronger trending effect of hierarchy in the overtrained relative to the moderately-trained group.

Exp 2d presented only C2 trials during the test phase. We expected that removing the need to dynamically retrieve the appropriate plan for the current context on each trial would reduce cognitive load and allow goal-directed control to dominate, making it more difficult to detect a habit effect. In this version of the task, since C1 trials were not available to contrast with C2 and compute the Habit Index, we examined how baseline-corrected slip error rates in C2 were influenced by overtraining. We found a trending effect of overtraining on C2 slips of action in overtrained participants in the early RT bin (*p* = .057). While the effect did not reach significance, it was more reliable than the effect of overtraining on the C2 baseline-corrected slip error rates in Exp 1a (*p* = .194), and comparable with that in Exp 1b (*p* = .089). Thus, we did not find support for our hypothesis that the design of Exp 2d would facilitate GD control and consequently weaken habit expression. Instead, the reduced overall cognitive control demand may have encouraged participants to relax GD control, strengthening habit expression in C2. This interpretation is supported by the high participant exclusion rate (63.3%) reflecting poor task engagement in Exp 2d (see Exclusion Criteria). Additionally, the effects of overtraining on HI in Exp 1a and Exp 1b (*p* = .011 and *p* = .040, respectively) were comparable with or more reliable than the effects of overtraining on slips of action in C2. This indicates that the propensity to make slip errors toward the more extensively trained context and the ability to avoid slip errors toward the less extensively trained context are both influenced by overtraining, highlighting the value of measuring both components to robustly detect overtraining-induced habits. Thus, while Exp 2d amplified the effect of overtraining on C2 slips of action, this came at the cost of poorer task engagement and the loss of the ability to contrast C2 with C1 behavior. This experiment also highlights the difficulty in predicting which manipulations decrease demand on GD control: while task-switching has been use to limit availability of GD control, the effect may be counterbalanced by participants actively recruiting more control when they need to switch.

Experiment 3 asked whether we can detect training-induced habit effect within-participant, by administering the test phase once after participants have completed the reversal phase and 200 training phase trials, then once more after they have completed 600 training phase trials in total. In this experiment, we did not observe a reliable effect of overtraining on the Habit Index in the early (*p* = .190) or late (*p* = .649) RT bin. We found that the Habit Index in the test phase block following 200 C1 trials (0.087, bootstrap CI [0.005, 0.143]) was slightly higher than that of the 200-trial group in Exp 1a (−0.026, [−0.078, 0.103]). Meanwhile, the Habit Index in the test phase block following 600 trials (0.130, [0.046, 0.179]) was similar to that of the 600-trial group in Exp 1a (0.139, [0.070, 0.210]). Thus, participants behaved more consistently with habitual control than expected after 200 trials of training, which led to a weaker effect of overtraining on HI.

## Discussion

Investigating habit formation in humans in controlled laboratory settings is notoriously challenging. Previous work has struggled to reliably (de Wit et al., 2018; Pool et al., 2022; Gera et al., 2023; de Wit et al., 2013; De Houwer et al., 2018) or overtly (Luque et al., 2020) demonstrate inflexible behaviors as a function of practice, which is critical to establishing that any inflexibility observed is mainly driven by a gradually-formed habit and not by individual trait- or state-level factors such as weak goal-directed control or fatigue. In the present study, we developed an experimental paradigm that satisfies this requirement: when tasked with flexibly switching between two sets of learned behaviors in Experiments 1a and 1b, overtrained participants were more biased than moderately-trained participants toward the more extensively practiced behaviors, at least at early (~0.5–1s) response times. At later (~2.5–3s) response times, this effect was largely abolished, showing that the effect observed at earlier response times is indeed driven by overtraining and not by other factors that vary with overtraining, such as weakened goal-directed control. This represents an advancement over some tasks previously used to study habits, which often appear to relate more strongly to measures of goal-directed control than to other behavioral or self-report measures of habits (Gillan et al., 2015; Friedel et al., 2014; Sjoerds et al., 2016; Linnebank et al., 2018). Our paradigm further improves upon existing habit tasks in two important ways. First, the overtraining procedure can be carried out within a single ~1-hour session consisting of several hundreds of iterations rather than thousands of iterations conducted over the course of several sessions across multiple days (Tricomi et al., 2009; de Wit et al., 2013; Hardwick et al., 2019); not only does our task design more closely reflect the number of repetitions required to form real-life habits Buyalskaya et al. (2023); Lally et al. (2010), but it also makes studying habits in the laboratory more feasible and accessible. Second, an effect of overtraining is found without imposing certain constraints, such as forced response times (Hardwick et al., 2019) or acute stress (Schwabe and Wolf, 2009), that poorly reflect the conditions under which habits emerge in naturalistic settings.

In addition to developing an efficacious, practical, and relatively naturalistic experimental paradigm for studying habit formation in the laboratory, we explored the roles of various task features that might contribute to the observation of an overtraining effect. The overtraining effect at early RTs disappeared when participants were given only one opportunity to respond (Exp 2a) and became less reliable when responding incorrectly incurred certain costs (Exp 1b), highlighting the importance of minimizing response inhibition for revealing habits. This is consistent with the Hardwick et al. (2019) model in which fast habitual response preparation, which grows faster with repeated practice, unfolds alongside a slower goal-directed response preparation process. In their case, habitual impulses were revealed in behavior by controlling the time delay after which participants were asked to respond. Our paradigm encourages habit expression by allowing participants unlimited response attempts over a long response period. Relatedly, since participants were free to respond as early or as late as they wanted, it was important not only to promote fast, uninhibited responses, but also to collect enough trials to compute sufficiently stable summary statistics of individual participants’ behavior. This point is reflected in the increased reliability of the overtraining effect in Exp 2b (computed based on 96 incongruent trials) relative to that in Exps 1a and 1b (48 incongruent trials).

Another key feature of our paradigm is the inclusion of both minimally practiced (C2) and more extensively practiced (C1) behaviors when testing for overtraining-induced behavioral inflexibility. Experiment 2d, which presented only C2 trials during the test phase, provided an opportunity to investigate how this feature impacts the ability to detect an overtraining effect. Rather than facilitating GD control and reducing behavioral inflexibility in C2 as we expected, the simplified task demands appeared to encourage participants to relax control, thereby amplifying inflexibility. However, the stronger overtraining effect on C2 action slips came at the cost of an untenable participant exclusion rate, highlighting a trade-off between minimizing response inhibition and maintaining task engagement. The design of Exp 2d parallels existing overtraining-based habit tasks, which typically train participants in an initial context for a controlled number of iterations, alter the task contingencies, then measure participants’ (in)flexibility in the altered context Tricomi et al. (2009); de Wit et al. (2013); Hardwick et al. (2019). One exception is Luque et al. (2020): during training blocks, participants learned that for each stimulus, the two available responses each led to an outcome worth 5, 10, or 100 points; in test blocks, one of the outcome types was devalued such that on some trials, the response previously leading to the more valuable outcome would now be worth 0 points. With longer training, participants under time pressure to respond within 500 ms did not exhibit the expected decrease in devaluation sensitivity in overt behavior, but they did exhibit increased reaction time (RT) switch costs, or average RTs for successful avoidance of devalued outcomes in test blocks (analogous to C2), relative to a baseline derived from the previous training block (without devalued outcomes, analagous to C1). In contrast to Luque et al. (2020), our test procedure induced an overtraining effect on overt behavior, likely owing in part to the relaxed response conditions outlined earlier. Incorporating both the original and altered contexts in the test phase enables the measurement of two complementary aspects of habitual behavior: susceptibility to errors favoring the extensively trained context, and ability to suppress errors favoring the minimally trained context. Both measures reflect the “pull” of more extensively practiced behaviors, and both increase with overtraining in our paradigm. One potential concern with assessing habit strength by combining these two measures, as we do with our Habit Index, is that the observed overtraining effects might be driven predominantly by the enhanced ability to avoid C2-consistent slips of action in C1 with overtraining (C1 component), and not by increased susceptibility to C1-consistent slips of action in C2 with overtraining (C2 component). Of the four task versions in which we detected a reliable overtraining effect (Exps 1a, 1b, 2b, and 2c), the reliability – assessed by the proportion of bootstrap samples showing an overtraining effect in the expected direction – of the C2 component exceeded that of the C1 component only in one experiment (Exp 1b). Nonetheless, consistently across all between-subject task versions, the majority of bootstrap samples exhibited the expected C2 overtraining effect (86.2% in Exp 1a, 90.1% in Exp 1b, 97.1% in Exp 2a, 96.7% in Exp 2b, 66.7% in Exp 2c, 82.1% in Exp 2d; see Fig. S1 and Table S1), which suggests that with larger sample sizes or more trials, the statistical reliability of the C2 component could be further improved. Importantly, the consistency of the C2 overtraining effect across experiments highlights its robustness as a component of habitual behavior.

In our paradigm, participants were required to select an abstract high-level plan at the beginning of a trial, then execute the plan across multiple decision-making steps. The need to hold a context-appropriate plan in mind within trials and flexibly switch between contexts across trials was a way to tax goal-directed control without applying pressure by e.g. imposing time constraints (Hardwick et al., 2019) or explicitly inducing stress (Schwabe and Wolf, 2009), encouraging behavior to naturally come under habitual control. Eliminating the hierarchical trial structure weakened, but did not abolish, the reliability of the overtraining effect in Experiment 2c, suggesting that hierarchy, while not strictly necessary for observing an overtraining effect, may facilitate habit expression under our paradigm. The results of Experiment 2b, where the degree of hierarchy was manipulated in a finer-grained manner within-participant, further support this interpretation. Relative to the 0-steps-hidden condition in which context information was available throughout the trial, the 2-steps-hidden condition, which required participants to maintain a mental representation of the current context, tended to enhance the Habit Index, especially in overtrained participants. While the robustness of this effect was not strongly supported by bootstrapping, it remains possible that concealing the context in stages 2–3 contributed to our ability to reliably detect overtraining effects in Experiments 1a and 1b. This hypothesis relates to previous work that found participants’ incorrect actions in a two-step task could be captured by a computational model that can select and learn the values of chunked action sequences alongside single actions; the authors argued that habits should be thought of as inflexible actions that emerge as elements of action sequences that come to be executed ballistically, with less reliance on environmental cues, through extended practice (Dezfouli et al., 2014). Our paradigm is similar to the two-step task in that responding correctly at later stages depends on successfully executing an appropriate multi-step plan. However, in our task, the selection of rigid action sequences is not an effective strategy, because the stage 3 stimulus (the maze arm the avatar ends up in) is unpredictable; thus, inflexible behavior on our task cannot be understood solely as the rigid execution of action sequences. Instead, we propose that habits correspond to the deployment of inappropriate hierarchical sub-policies, similar to “options” in hierarchical reinforcement learning (Botvinick et al., 2009; Xia and Collins, 2021; Li and Collins, 2025): the inflexible actions we observe at stage 3 may arise from selecting the appropriate stimulus-action policy for the cued context at stage 1, but being attracted toward the more extensively practiced C1 policy in the course of rolling out the initially-selected policy. This idea could be tested in future work by exploring the space of hierarchical task designs. For instance, if inflexibility arises from inappropriately switching policies during the rollout of an initially-selected policy, increasing the overlap between the two policies, for example by increasing the number of intermediate states with the same correct response, should amplify the influence of the C1 policy, particularly in overtrained participants. In addition to confirming various predictions of the high-level idea that policy confusion leads to the inflexibility observed under our hierarchical task design, future work could use computational models to formalize the cognitive processes underlying habit formation and expression.

Our findings come with a few limitations – the first being that the current within-participant design (Exp 3) is not able to induce a reliable effect of training duration on the Habit Index. This suggests that some aspect of Exp 3 causes participants to behave in a manner consistent with habits after only 200 trials of training. One candidate explanation is that the ratio of incongruent to congruent trials in the test phases of Exp 3 is twice that in Exp 1a (see Experimental Procedure), which may have made it challenging to behave in a goal-directed manner even during the first test phase; however, the same is true of Exp 2b, in which a robust overtraining effect was observed, making this explanation less likely. Another possible explanation is that the response windows were shortened from 3s to 2.5s in Exp 3 to keep overall experiment time within one hour. The stronger time constraint may have encouraged participants to speed their responses in the first test phase, resulting in more habitual slips of action, and thus weakening moderately- vs. overtrained comparisons. This explanation is supported by RT data: one-tailed Wilcoxon rank-sum tests comparing participants’ median first RTs in incongruent arms following 200 training trials in Exp 1a and Exp 3 revealed significantly slower responses in Exp 1a for both contexts (C1 median RT = 0.96s for Exp 1a and 0.75s for Exp 3, *W* = 938, Bonferroni corrected *p* = .0012; C2 median RT = 0.90s for Exp 1a and 0.74s for Exp 3, *W* = 939, Bonferroni corrected *p* = .0013; see also Figure S3 and Table S1). Another possible contributor is that in Exp 3, overtraining is confounded with test phase experience. Indeed, participants tended to make fewer slip errors in the second half than in the first half of the first test phase, especially in C2 (Figure S5), raising the possibility that we would have observed a stronger HI following 600 trials of training had participants not experienced the first test phase. While this limitation is a target for future research, we do not think it undermines the usability of the current protocol, as the between-participant versions of the task have been shown to reliably provide measures of both habit formation (HI in early time bins) and control (decrease in HI from early to late time bins).

A second limitation of our paradigm is the relatively high participant exclusion rate. This problem was somewhat mitigated in Experiment 1b, which introduced a game mechanic that weakly penalized incorrect responses, discouraging the use of satisficing strategies and reducing the exclusion rate from ~50% in Experiment 1a to a more standard ~30%. It should be noted, however, that this improvement came at the cost of a slight reduction in the reliability of the overtraining effect. A useful avenue for future work would be to explore different ways to encourage participants to respond freely while discouraging “cheating” strategies, without over-engineering the task with features that compromise ecological validity.

A final and perhaps the most substantial caveat we offer is that the paradigm has not yet been evaluated with respect to its convergent validity with other habit induction tasks, or its external validity in capturing individual differences in self-reported or behavioral measures of real-life habits or relevant mental health symptoms. Establishing these would significantly strengthen the present results and interpretations. However, we believe the ability to identify habit-like behavior in a way that is separable from control (rather than a trade-off, as is typical), offers a promising direction.

In closing, the present work introduces an experimental paradigm that can induce and reveal inflexible responding in human participants within a single ~1-hour session, without imposing artificial constraints such as extreme time pressure or explicitly induced stress. Crucially, behavioral inflexibility at early response times is reliably amplified with extended practice, while later responses are largely unaffected by practice, indicating that the patterns observed at early response times reflect gradually-formed habits rather than individual differences in goal-directed control. Several task features contribute to the robustness of the critical overtraining effect, including the allowance for multiple response attempts, a hierarchical multi-step trial structure, and a mix of extensively and minimally practiced contexts when testing for habits. This paradigm offers a reliable, practical, and ecologically valid way to investigate habit formation and expression in human subjects under controlled laboratory conditions. It holds promise for advancing our understanding of the cognitive and neural mechanisms underlying both the gradual transition from predominantly goal-directed to predominantly habitual control and how the two systems dynamically interact and compete at shorter timescales. It also provides a tool that can be adapted to study individual differences in both adaptive and maladaptive habits, offering insights into the processes that support healthy behaviors and those that contribute to conditions like addiction, OCD, and obesity.

## Methods

### Participants

All experiments were administered online to undergraduate students from the University of California, Berkeley, recruited through the UC Berkeley Research Participation Program (RPP); students received course credit for their participation. Some experiments also recruited participants through Prolific who received monetary compensation for their participation.

160 RPP participants (118 female, 40 male, 2 other; age: *mean*=21.1, *sd*=3.40, *min*=18, *max*=43) completed Experiment 1a. Of these, 17 participants were excluded based on self-reported exclusion criteria – namely, for indicating in their post-task survey that they used external aids (e.g. post-it notes on the computer screen) to perform better on the task, or that they believed their data should be excluded from analysis – leaving 143 participants.

109 RPP participants (80 female, 25 male, 4 other / declined to answer; age: *mean*=20.6, *sd*=2.19, *min*=18, *max*=32) and 41 Prolific participants (14 female, 26 male, 1 other; age: *mean*=34.5, *sd*=12.8, *min*=20, *max*=67) completed Experiment 1b. Of these, 18 were excluded based on self-reported exclusion criteria, leaving 132 participants (91 RPP, 41 Prolific).

159 RPP participants (110 female, 45 male, 4 other/ declined to answer; age: *mean*=21.5, *sd*=4.32, *min*=18, *max*=54) completed Experiment 2a. Of these, 29 were excluded based on self-reported exclusion criteria, leaving 130 participants.

242 RPP participants (173 female, 65 male, 4 other / declined to answer; age: *mean*=20.6, *sd*=2.25, *min*=18, *max*=36) completed Experiment 2b. Of these, 69 were excluded based on self-reported exclusion criteria, leaving 173 participants.

103 RPP participants (82 female, 19 male, 2 other / declined to answer) and 41 Prolific participants (21 female, 19 male, 1 declined to answer) completed Experiment 2c. Of these, 9 were excluded based on self-reported exclusion criteria, leaving 135 participants (95 RPP, 40 Prolific).

152 RPP participants (116 female, 32 male, 6 declined to answer) and 39 Prolific participants (16 female, 22 male, 1 other) completed Experiment 2d. Of these, 20 were excluded based on self-reported exclusion criteria, leaving 135 participants (132 RPP, 39 Prolific).

66 RPP participants (51 female, 15 male; age: *mean*=20.4, *sd*=2.42, *min*=18, *max*=35) completed Experiment 3. Of these, 5 were excluded based on self-reported exclusion criteria, leaving 61 participants.

For all experiments, additional participants were excluded on the basis of behavioral indices that suggested they were not fully engaging with the task (see Exclusion Criteria).

### Exclusion Criteria

We applied the following exclusion criteria to identify careless or inattentive participants: 1) more than five missed trials during either the training (C1) or reversal (C2) phase (indicating low engagement in learning the associations), 2) failure to meet the C2 training criterion (correct first response in four out of five most recent exposures to each maze location) within 100 trials (indicating failure to learn C2, making C2 test phase errors uninterpretable), 3) fewer than two responses on more than 25% of trials in any context and congruent/incongruent arm type despite instructions to respond more than once during the test phase (indicating a failure to understand the multi-response design of test phase trials), and 4) evidence of satisficing strategies in the test phase (indicating failure to engage with the task in good faith, and undermining interpretability). Satisficing behaviors included random spamming (more than 1 unique key press in the last five responses on at least half of trials in both congruent and incongruent arms), strategic spamming (more than 1 unique key press in the last five responses on at least half of trials in incongruent arms, but not in congruent arms; typically such participants alternated between the two reversed keys), and making reliable errors (all trials ending on an incorrect response for any context and maze arm). In participants who passed all other exclusion criteria, including the C2 training criterion, these satisficing behaviors (see Fig. S4 for examples of each) point to a failure to apply knowledge acquired in the training and reversal phases during the test phase. In the case of Exp 2a, since only one response was allowed in test phase trials, the exclusion criteria for spamming responses during the test phase were not applied, and participants were excluded for making no response (instead of < 2 responses) in 25% of trials in any context and congruent/incongruent arm type.

Table 5 shows the proportion of participants excluded by each of these criteria in each version of the experiment. Across tasks versions, a considerable proportion of participants is excluded for failure to adequately learn C2 within the allotted number of trials, and for satisficing behaviors during the test phase (see Table S2 for a summary of behavioral results when satisficing-related exclusions were not applied).

### Experimental Procedure

#### Experiment 1a

##### Learning phase.

For each of six states S1-S6 (corresponding to six locations with respect to a cross-shaped maze: outside the maze, the maze center, and the top, right, bottom, and left arms of the maze), participants learned to select the correct action from six available key presses A1-A6 (corresponding to keys S, D, F, J, K, and L, assigned randomly). Participants were instructed that they would press keys to control a stick figure avatar named Alex in order to collect stars from the maze. At the beginning of each trial, Alex appeared in a starting position outside of the maze (S1). Correctly selecting A1 in S1 moved Alex to the center of the maze (S2). Correctly selecting A2 from S2 moved Alex stochastically to one of the maze arms (S3-S6). Correctly selecting A3-A6 in response to S3-S6, respectively, resulted in a one-point star reward, followed by a 0.5 second inter-trial interval, after which the next trial began with the presentation of S1. Alex’s color was only visible when outside of the maze (the first stage of each trial), and changed to black when in the center or arms of the maze (second and third stages). Two seconds were allotted for each stage within a trial. Timing out of any stage prompted the participant to choose an action faster, and selecting the incorrect action at any stage prompted the participant to retry the stage. Participants were given nine attempts to advance from each stage; failure to respond correctly within nine attempts would abort the trial and proceed to the first stage of the next trial. Each block of trials was constructed pseudorandomly by concatenating 25 shuffled sequences of four trials each terminating one of the four maze arms, resulting in 100 trials per block. Participants completed either two or six blocks in the original learning context, Context 1 (C1), depending on whether they had been randomly assigned to the moderately-trained group or the over-trained group. Participants were given breaks, up to 2 minutes long, in between blocks. During breaks, participants were informed how well they performed relative to others (“It took you X presses to get to the star on average in the past 100 trials. This means you did better than Y% of players in this part of the game”) to encourage continued engagement in the task. Participants also completed eight practice trials after reading the task instructions and before beginning the first learning block, using a different set of keys: W, E, R, I, U, and O, assigned randomly to A1-A6.

##### Reversal phase.

Following the learning phase, participants were introduced to Context 2 (C2), which was similar to C1, except that the correct responses for two of the maze arms were swapped. Participants were instructed that they would control a new avatar named Cameron and that the correct key presses might differ from those learned in C1. As in C1, Cameron’s color, which was different from Alex’s color, was only visible when outside the maze. Participants trained in C2 for one block, constructed pseudorandomly as in the learning phase; the full block contained 100 trials but was cut short if achieving an accuracy criterion of correct response at first attempt in four out of the five most recent exposures to each of S1-S6.

##### Test phase.

In the final phase of the experiment, participants were presented with 48 C1 and 48 C2 trials, randomly interleaved. The trial sequence was constructed pseudorandomly by concatenating 12 shuffled sequences of four C1 trials and four C2 trials. As in the learning and reversal phases, the context information (C1/C2 avatar color) was only available in the first stage of each trial, requiring participants to hold this information in mind to guide action selection in the third stage. Unlike in the learning and reversal phases, during which a valid response would immediately result in feedback, participants were allowed to make multiple key presses to collect points from the maze arm for 3 seconds during the last stage of each trial. The number of stars collected was displayed following the 3-second response period only during the eight practice trials preceding the test phase; the actual test phase was conducted in extinction (without reward feedback) to prevent further learning; participants were instructed that they would find out how many stars they collected at the end of the experiment. Before the test phase practice trials, participants were also given an opportunity to remember how to control each avatar. This memory refresher consisted of two repetitions of the following: up to eight C1 learning phase trials (fewer if reaching a criterion of correct response at first attempt in one out of two exposures to each state), followed by up to eight C2 reversal phase trials (fewer if reaching criterion), with feedback.

#### Experiment 1b

Experiment 1b introduced tweaks on 1a in an effort to boost participant engagement and improve exclusion rates. Animations were added when presenting stimuli and feedback for a more gamified experience. Less superficially, during training and reversal, the feedback messages (“Try again!”) for incorrect stage 3 responses were replaced by a game mechanic wherein each incorrect response earned one “anti-star” (a black circle with a star cut out from the center), each of which could cancel out one star. Anti-stars accumulated at the right side of the screen on each trial. When a participant selected the correct stage 3 response after having collected one or more anti-stars, one anti-star would become filled with a yellow star, then the star and anti-star would fade, resulting in no points earned. When a participant made the correct stage 3 response on their first attempt, a yellow star would appear, then fade while moving to the top of the screen, where the point count for the current block was incremented by one point. Thus, during the training and reversal phases, participants only won points for correct first attempts in stage 3. In the eight practice trials preceding the test phase, the number of stars and anti-stars collected were displayed following the 3-second response period; stars canceled out by anti-stars faded, leaving any remaining stars along with a message stating how many stars were collected. As in Experiment 1a, participants were informed that they would earn stars in the same way during the actual test phase, but that they would not be shown how many stars (or anti-stars) they collected. This mechanic was intended to allow participants to respond freely during the test phase while discouraging them from using satisficing strategies to earn points without fully engaging with the task.

#### Experiment 2a

Experiment 2a was identical to Experiment 1a, with the exception that only one response was allowed during the last stage of each trial. The task proceeded to the next trial once a valid response was selected or the 3-second response period expired. Participants were informed whether they got a star following each of the eight practice trials; the actual test phase was conducted in extinction.

#### Experiment 2b

Experiment 2b was similar to Experiment 1a, with a within-participant manipulation to the level of hierarchy, or the number of steps over which participants were obliged to keep in mind the current decision-making context, in the test phase. The test phase included full-hierarchy trials, in which C1/C2 context information (the color of the avatar) was only visible in the first stage of a trial; intermediate-hierarchy trials, in which context information was available until the second stage; and minimal-hierarchy trials, in which context information was available throughout all three stages. Following the memory refresher and practice test phase trials, participants were instructed that in some trials, parts of the maze would be revealed so that they would able to see the avatar’s identity in addition to its location. To accommodate the hierarchy manipulation while keeping the test phase reasonably short, incongruent maze arms were presented twice as many times as consistently-mapped arms. The test phase trial sequence was constructed pseudorandomly by concatenating four shuffled sequences, each consisting of six (one presentation of consistently-mapped arms and two presentations of reversed maze arms) C1 trials for each hierarchy level and similarly for C2, resulting in 144 test phase trials. A break of up to 2 minutes was given after the first 72 trials of the test phase in order to lessen fatigue.

#### Experiment 2c

Experiment 2c was similar to Experiment 1a, but participants were trained and tested only on the arms of the maze – that is, in stage 3 only.

#### Experiment 2d

Experiment 2c was identical to Experiment 1a, with the exception that participants were tested in context C2 only. The memory refresher and practice test phase trials included both contexts.

#### Experiment 3

Experiment 3 was similar to Experiment 1, but the training duration manipulation was conducted within-participant instead of between moderately-trained and over-trained participant groups. All participants completed two test phase blocks: the first after two blocks of training in C1, and the second after four additional blocks of training in C1. To minimize biases that would be introduced by collecting the first, but not the second, test block immediately following C2 training, participants trained to criterion in C2 before beginning the first C1 learning phase block. In an effort to keep the task duration under one hour, the two maze arms whose state-action mappings were reversed from C2 to C1 were each presented twice as many times as the consistently-mapped maze arms. The test blocks were constructed pseudorandomly by concatenating six shuffled sequences of six C1 trials (one presentation of the consistently-mapped arms and two presentations of the incongruent arms) and six C2 trials (similar), resulting in 72 trials per test block. The 3-second period allotted to the third stage of each test phase trial was also shortened to 2.5 seconds. Additionally, test phase instructions and practice trials were only given before the first test phase block; memory refresher trials were given prior to the practice trials preceding the first test block, and again right before the second test block. The number of stars collected was displayed following the 2.5-second response period only during the practice trials preceding the first test block; the actual test blocks were conducted in extinction.

### Analyses

#### Habit Index

To measure the strength of each participant’s habit, we focused on test phase responses to incongruent maze arms; these were either correct responses, slip errors (responses that would have been correct for the opposite context), or random errors (any other incorrect response). When control is more habitual, we would expect 1) more slips of action in C2 toward the more-extensively trained C1, reflecting increased inflexibility with practice, and 2) fewer slips of action in C1 toward the less-extensively trained C2, reflecting increased fluency with practice. We quantified and aggregated both tendencies by contrasting the proportion of responses that are slips of action in C2 with that in C1.

Since slip errors could arise from random responding as well as from slips of action toward the opposite context, we estimated for each context the proportion of responses attributable to slips of action by subtracting out a baseline random error rate from the slip error rate. Of the six responses available to the participant, four are associated with terminal maze arms; since each incongruent maze arm had one terminal response that was a slip error and two terminal responses that were random errors, we estimated the baseline random error rate per arm as $P_{\mathrm{random}}/2$.

Thus, we defined a Habit Index (HI) that measures how strongly behavior is biased toward the more extensively practiced context and away from the less-extensively trained context, while accounting for the possibility that some slip errors may be driven by an individual’s propensity to mix up the two contexts due to weak goal-directed control, lapses in attention, or other factors:

(2) $$HI=\left(P_{\mathrm{slip}}^{C2}-P_{\mathrm{random}}^{C2}/2\right)-\left(P_{\mathrm{slip}}^{C1}-P_{\mathrm{random}}^{C1}/2\right)$$

For each participant, we computed HI within evenly-spaced time bins spanning the interval from 0.5s to the end of the 3s free response period. Responses before 0.5s were excluded from this analysis due to noise (for instance, in Exp 1b, 58.7% of participants made zero responses before 0.5s in incongruent arms in C2; see Figure S3).

If the habitual system quickly prepares a frequently-practiced response, which then gets overridden by goal-directed cognitive control Hardwick et al. (2019), HI should be positive in earlier time bins, then fall toward zero in later time bins. If habitual control is expressed more strongly in over-trained participants, HI in early time bins should be higher in over-trained participants than in moderately-trained participants. If over-trained participants learn the C2 contingencies as well as moderately-trained participants, and are able to exert goal-directed control to a similar degree as moderately-trained participants, HI should fall to a similar level in over-trained and moderately-trained participants in later time bins.

#### Bootstrapping Analysis

We performed a bootstrapping analysis to evaluate the reliability of the effect of overtraining on the Habit Index and its components in each version of the task. To generate each bootstrap sample, *N* = 35 participants were sampled with replacement separately from each group (600-trial and 200-trial groups); each sampled participant’s trials were next resampled with replacement within each context (C1, C2), ensuring the original trial structure was preserved. For each bootstrap sample, we computed P(slip) for each participant in each context and each 0.5s time bin, by averaging across trials the proportions of incongruent arm responses falling in each time bin and context that were slip errors, and similarly for P(rand) and random errors; the average proportions were used to compute P(slip)−P(rand)/2. We computed the contrast *C*2 − *C*1 for each measure, and the difference between early (0.5–1s) and late (2.5–3s) RT bins for each level of context (including the contrast *C*2 − *C*1). From each bootstrap sample, we generated a null distribution sample by swapping the group labels for a random half of participants from each group.

We applied the following steps separately to the original bootstrap sample and to the null distribution sample with shuffled group labels. We first averaged each measure, including contrasts, across participants for each group. Only participants with responses falling in a given RT bin in least 5 trials contributed to the group average measure for that RT bin (the smaller number of trials was taken when counting the number of response-containing trials for a contrast measure). For example, if P(slip) of a 200-trial group participant RT bin (0.5,1] in C1 was computed by averaging 5 C1 trials, and the same measure in C2 was computed by averaging 4 C2 trials, the participant’s P(slip) would contribute to the 200-trial group’s average P(slip) for C1, but not for C1 or *C*2 − *C*1. Finally, using the average measures computed for each group, we computed the difference between the 600-trial and the 200-trial group. This was repeated for 10000 bootstrap iterations. The proportion of bootstrap samples in which a given contrast was in the expected direction (e.g., the proportion of samples in which $P_{\mathrm{slip}}^{C2}-P_{\mathrm{slip}}^{C1}>0$) was calculated to assess the reliability of specific effects. The proportion of null distribution samples in which the statistic of interest exceeded the empirical value observed in the full dataset was calculated to evaluate the likelihood of observing the empirical result under the null hypothesis.

For some experiments, the bootstrapping procedure differed slightly from that outlined above. For Exp 2a, rather than absolute RT bins, responses were sorted into an early bin if occurring before the participant-wide median RT (the median of all participants’ median RTs in incongruent trials), and a late bin otherwise. For Exp 2b, separate two null distributions were generated: one with shuffled group labels, representing a null hypothesis that the observed differences between the 600-trial and 200-trial groups were due to random chance, and another with the 0-step-hidden and 2-step-hidden hierarchy labels swapped for a random half of participants, representing a null hypothesis that any effect of hierarchy arose from unsystematic, participant-specific variability rather than systematic effects of the hierarchy manipulation; the difference between 2-step and 0-step hidden conditions was computed along with the context and RT contrasts. For Exp 2c, since C1 was not included in the test phase, bootstrapping tested for effects on measures involving only $C2:P_{slip}^{C2},P_{\mathrm{rand}}^{C2}$, and $P_{slip}^{C2}-P_{\mathrm{rand}}^{C2}/2$. For Exp 3, since the training duration manipulation was conducted within-participant, the 600-trial minus 200-trial contrast was computed after swapping the test block labels (first test following 200 trials / second test following 600 trials); unlike Exps 1–2, the training duration contrast was computed within-participant along with the context and RT contrasts before averaging across participants, rather than after averaging after participants in each group.

#### Regression analysis

To supplement our analysis of the HI summary statistic within fixed time bins, we analyzed behavior at the level of individual responses by fitting a multinomial regression model predicting whether test phase responses in incongruent arms are slip errors, random errors, or correct responses as a function of amount of training (across participant groups), response time (within participant), and context (within participant): responseType ~ context * group * RT + (1 | id). The model was estimated using all responses on incongruent trials regardless of RT, not just those occurring after 0.5s which was the RT cutoff in RT bin based analyses. The model used a multinomial logit link and included random intercepts for participants to account for individual differences in propensity to make slip errors and random errors. Model comparison via 10-fold cross-validation using loo_compare determined that a model with random intercepts only had a higher expected log predictive density (ELPD) compared to models including random slopes for context, RT, or both. The outcome variable, *responseType*, was categorical, with slip errors and random errors modeled relative to correct responses as the baseline category. Predictors were coded as follows: context (−1 for C1, +1 for C2), group (−1 for the 200-trial group, +1 for the 600-trial group), and RT (z-scored). The model estimated coefficients for two comparisons: the log-odds of a response being a slip error versus the baseline category (correct response), and the log-odds of a response being a random error versus the baseline category. This allowed us to quantify how the predictors and their interactions influenced the likelihood of each error type relative to correct responses.

We fit the model using the brms R package, which interfaces with Stan (Gelman et al., 2015) to estimate the posterior distribution of regression model parameters via Markov Chain Monte Carlo (MCMC). Sampling was performed with four MCMC chains, each with 4000 iterations, of which 3000 were retained after warmup, resulting in 12000 posterior draws; priors were weakly informative.

Following sampling, we tested hypotheses about how variables influence the probabilities of slip errors, random errors, and baseline-corrected slip error probability. Specifically, we tested whether the extent to which the measures of interest were larger in C2 relative to C1 increased with overtraining, at an early RT (0.5s) and at a late RT (3s). Using the hypothesis function in brms, the posterior samples of the regression coefficients were used to compute posterior distributions of the predicted probabilities of slip errors and random errors by inverse logit transforming the linear predictor for each combination of context and group, at early (0.5s) and at late (3s) RTs. From the predicted probabilities, we also computed the predicted baseline-corrected slip error rate. This allowed us to test whether the C2 minus C1 difference in each measure (slip error probability, random error probability, and baseline-corrected slip rate) was reliably larger in the 600-trial group vs. the 200-trial group at early and late RTs.

#### Custom Hierarchical Bayesian Model

Using Bayesian hierarchical modeling methods, we fit a custom probabilistic model accounting for the generation of stage 3 response types (correct, slip errors, or random errors) in the test phase as a mixture of two distinct strategies: a random strategy and an on-task strategy. The model was estimated using all responses, regardless of RT, on both congruent and incongruent trials.

The model was designed to jointly estimate the probability of engaging in the random strategy, and the probability of producing slip errors under the on-task strategy (i.e., slips of action), as a function of context (C1, coded as −1, or C2, coded as +1), training group (200 trials, coded as −1, or 600 trials, coded as +1), response time (RT, z-scored), and trial type (congruent, coded as −1, or incongruent, coded as +1).

To estimate the probability of slips of action, the model estimated fixed effect coefficients $\beta 0_{\mathrm{slipErr}}^{\mathrm{TaskStrat}},\ldots ,\beta 16_{\mathrm{slipErr}}^{\mathrm{TaskStrat}}$, including a random intercept, capturing shared variability in participants’ general propensity to make slip errors under the on-task strategy:

(3) $$logit\left(P_{slipErr}^{\mathrm{TaskStrat}}(X)\right)=z_{\mathrm{slipErr}}^{\mathrm{TaskStrat}}(X)=\beta 0_{\mathrm{slipErr}}^{\mathrm{TaskStrat}}+\beta 1_{\mathrm{slipErr}}^{\mathrm{TaskStrat}}X1+\ldots +\beta 16_{\mathrm{slipErr}}^{\mathrm{TaskStrat}}X16$$

where X=(X1,…,X16) are the predictor variables: context, training group, response time, congruent/incongruent trial type, and their interactions. The model also estimated random effect coefficients capturing participant-specific variability in propensity to respond at random:

(4) $$\begin{array}{l} \mathrm{logit}\left(P_{\mathrm{slipErr}\;}^{\mathrm{TaskStrat}\;}\left(X\right)\left[id\right]\right)=z_{\mathrm{slipErr}\;}^{\mathrm{TaskStrat}\;}\left(X\right)+b0_{\mathrm{slipErr}\;}^{\mathrm{TaskStrat}\;}\left[id\right]+b1_{\mathrm{slipErr}\;}^{\mathrm{TaskStrat}\;}X1+\\ \;\;\ldots +b8_{\mathrm{slipErr}\;}^{\mathrm{taskStrat}\;}\left[id\right]X8 \end{array}$$

where “id” indexes the participant and $z_{\mathrm{slipErr}}^{\mathrm{TaskStrat}}(X)$ is the linear predictor from the fixed effects and predictor variables. Note that there are only 8 random effect predictors, rather than 16: since training group was constant within-participant, group and its interactions were excluded from the random effects structure.

The model similarly estimated coefficients capturing overall and participant-specific variability in random strategy use, yielding $P_{\mathrm{randStrat}}(X)[id]$. The model also estimated for each participant a fixed constant $P_{\mathrm{resp}}^{\mathrm{RandStrat}}[id]$ governing the probability of each response type under the random strategy, modeled as

(5) $$P_{resp}^{\mathrm{RandStrat}}[id]=1/6+(1/4-1/6)\times {\mathrm{logit}}^{-1}\left(\beta_{\mathrm{resp}}^{\mathrm{RandStrat}}+b_{\mathrm{resp}}^{\mathrm{RandStrat}}[id]\right)$$

where $\beta_{\mathrm{resp}}^{\mathrm{RandStrat}}$ is a fixed intercept, and $b_{\mathrm{resp}}^{\mathrm{RandStrat}}[id]$ is the participant-specific random intercept. The inverse logit function, ${\mathrm{logit}}^{-}1$, maps the linear predictor onto the probability scale between 0 and 1, the result of which is scaled to be between the lower and upper bounds 1*/*6 and 1*/*4, accounting for the possibility that some participants may respond randomly using all 6 available keys, while others may respond using the 4 keys associated with the terminal maze arms.

The model predicted the probability of each type of response by combining the above components. Under the on-task strategy, in incongruent arms, the probability of the slip error response was $P_{\mathrm{slipErr}}^{\mathrm{TaskStrat}}(X)[id]$, the probability of the correct response was ${1-P}_{\mathrm{slipErr}}^{\mathrm{TaskStrat}}(X)[id]$, and the probability of random errors was 0; in congruent arms, the probability of a correct response was 1, and the probability of an error was 0. Under the random strategy, in incongruent arms, the probability of the correct response and the probability of the slip error response were each $P_{\mathrm{resp}}^{\mathrm{RandStrat}}[id]$, and the probability of a random response was $1-2\times P_{\mathrm{resp}}^{\mathrm{RandStrat}}[id]$; in congruent arms, the probability of the correct response was $P_{\mathrm{resp}}^{\mathrm{RandStrat}}[id]$, and the probability of an error was $1-P_{\mathrm{resp}}^{\mathrm{RandStrat}}[id]$.

The probabilities of each response type under the two strategies were combined using $P_{\mathrm{randStrat}}(X)[id]$ to weight the response type probabilities under the random strategy, and $1-P_{\mathrm{randStrat}}(X)[id]$ to weight the response type probabilities under the on-task strategy. For incongruent trials, the three response types (correct, slip error, random error) were modeled using a category distribution; for congruent trials, the two possible response types (correct, error) were modeled using a Bernoulli distribution.

The model was coded in the Stan programming language. To guide the sampling process, priors were specified for each parameter in the model. Normal priors with a mean of 0 and a standard deviation of 1.5 were assigned to the fixed effect coefficients β. The standard deviations of the random effects were modeled with half-Cauchy distributions, using a scale parameter of 1, and an upper bound at 10. Correlations between all random effects (except for the random effects governing $P_{\mathrm{resp}}^{\mathrm{RandStrat}}[id]$) were included in the model; the correlation matrix was parameterized using a Cholesky decomposition, using the LKJ prior with a shape parameter of 1, which treats all possible correlation matrices as equally likely a priori.

The model was run with four MCMC chains, each consisting of 4000 iterations; of these, the first 1000 were used as warmup iterations, leaving 3000 posterior samples per chain following sampling. The adapt_delta parameter was set to 0.99, aimed at reducing the number of divergent transitions during sampling. To ensure that the sampler succeeded in fully exploring the parameter space and converged to a stable posterior distribution, we ensured that no divergent transitions were encountered, that effective sample size was at least 400 (100 times the number of chains) for all parameters, that the $\overset{ˆ}{R}$ diagnostic did not exceed 1.01 for any parameters, and that the diagnostic metric E-BFMI≥ 0.2 for all chains (Baribault et al., 2023).

We passed all posterior samples of the fixed effects and fixed intercepts to the model to obtain posterior samples of the predicted probability of engaging in the random strategy and predicted probability of slips of action under the on-task strategy at different settings of the task variables (context, RT, trial type, and group). We visualized the average and 90% credible intervals of the probabilities, and measured the proportion of posterior samples for which the difference between C2 and C1 in the probability of on-task slips of action was more positive for the 600-trial group than for the 200-trial group, at *RT* = 0.5*s* and at *RT* = 3*s*.

## Figures and Tables

**Table 1:** Summary of task features included in each version of the experiment.

	Task features					
Task Version	>1 response allowed in test phase	Test phase context observability	3-stage trial structure	Test phase context switching	Between-participant	Additional gamification
Exp 1a	✓	✓	✓	✓	✓	
Exp 1b	✓	✓	✓	✓	✓	✓
Exp 2a		✓	✓	✓	✓	
Exp 2b	✓		✓	✓	✓	
Exp 2c	✓	✓		✓	✓	
Exp 2d	✓	✓	✓		✓	
Exp 3	✓	✓	✓	✓		

**Table 2:** Summary of bootstrap analysis computing various contrasts between groups, RT bins, and contexts of the slip error rate, random error rate, and baseline-corrected slip error rate. Column “N ” contains the average (across bootstrap iterations) number of bootstrapped participants contributing to the measure (bootstrapped participants must have five or more responses in a given RT bin and context in order to contribute to the aggregate measure for that bootstrap iteration; for contrasts between groups, the number of participants in the smaller group was taken; see Methods). Included in the table are the 95% confidence intervals of each measure, the proportion of bootstrap samples greater than 0, and the significance of the difference measured in the full dataset with respect to a null distribution generated from bootstrap samples with shuffled group labels. Bold text highlights the main contrast of interest: the effect of overtraining on HI in the early RT bin.

group	context	RT bin (s)	N	measure	empirical	.95CI	%>0	*p*_null>emp._

600	C2-C1	early (0.5,1]	25.3	P(slip)	0.144	[.052,.245]	99.5	
				P(rand)	−0.002	[−.036,.040]	48.1	
				P(slip)-P(rand)/2	0.145	[.048,.250]	99.4	

200	C2-C1	early (0.5,1]	23.5	P(slip)	−0.017	[−.117,.078]	38.8	
				P(rand)	−0.014	[−.073,.036]	29.7	
				P(slip)-P(rand)/2	−0.009	[−.112,.093]	44.6	

600	C2-C1	early-late (0.5,1]-(2.5,3]	25.3	P(slip)	0.100	[.023,.184]	98.4	
				P(rand)	−0.007	[−.040,.033]	41.5	
				P(slip)-P(rand)/2	0.103	[.021,.193]	98.0	

200	C2-C1	early-late (0.5,1]-(2.5,3]	23.5	P(slip)	−0.156	[−.100,.075]	42.4	
				P(rand)	−0.005	[−.073,.050]	40.6	
				P(slip)-P(rand)/2	−0.013	[−.104,.093]	46.1	

**600–200**	**C2-C1**	**early (0.5,1]**	**22.7**	P(slip)	0.160	[.028,.301]	97.8	.029
				P(rand)	0.012	[−.048,.087]	66.2	.385
				**P(slip)-P(rand)/2**	**0.154**	**[.010,.299]**	**96.3**	**.040**

600–200	C2	early (0.5,1]	25.8	P(slip)	0.071	[−.031,.173]	86.8	.132
				P(rand)	−0.034	[−.084,.018]	14.4	.866
				P(slip)-P(rand)/2	0.088	[−.018,.191]	91.1	.089

600–200	−C1	early (0.5,1]	27.5	P(slip)	0.064	[−.007,.153]	93.0	.089
				P(rand)	0.045	[−.004,.105]	93.8	.085
				P(slip)-P(rand)/2	0.041	[−.037,.134]	82.1	.201

600–200	C2-C1	late (2.5,3]	34.2	P(slip)	0.023	[−.028,.077]	75.8	.235
				P(rand)	0.004	[−.025,.032]	59.9	.411
				P(slip)-P(rand)/2	0.021	[−.031,.076]	73.7	.260

600–200	C2-C1	early-late (0.5,1]-(2.5,3]	22.2	P(slip)	0.115	[−.006,.234]	94.1	.054
				P(rand)	−0.002	[−.066,.081]	54.7	.533
				P(slip)-P(rand)/2	0.116	[−.021,.242]	91.6	.072

**Table 3:** Results of hypothesis testing applied to constrasts (C2 minus C1, 600-trial group minus 200-trial group) of predicted probabilities obtained from posterior samples of the multinomial regression model’s fixed effect coefficients. Included in the table for each measure and RT is the mean (“Estimate”), standard deviation, and 90% credible interval of the resulting distribution, as well as the evidence ratio (of the number of posterior samples for which the contrast was positive, providing evidence for the hypothesis, to the number of samples for which the contrast was negative, providing evidence against the hypothesis) and the posterior probability (the proportion of samples for which the contrast was positive). Bold text highlights the main contrast of interest: the effect of overtraining on predicted HI in the early RT bin.

measure	RT (s)	Estimate	SD	90% CrI	Evidence Ratio	Posterior Prob.

P(slip)	0.5	0.172	.029	[.127,.221]	Inf	1.00*
	3	0.003	.004	[−.004,.009]	3.54	0.78

P(rand)	0.5	0.001	.007	[−.009,.012]	1.45	0.59
	3	0.001	.001	[−.001,.003]	3.31	0.77

**P(slip)-P(rand)/2**	**0.5**	**0.154**	**.029**	**[.109,.204]**	**Inf**	1.00*
	3	−0.001	.004	[−.008,.006]	0.71	0.41

**Table 4:** Similar to Table 3, results of hypothesis testing applied to constrasts (C2 minus C1, 600-trial group minus 200-trial group) of predicted probabilities of slips of action under the on-task strategy, obtained from posterior samples of the custom Stan model.

measure	RT (s)	Estimate	SD	90% CrI	Evidence Ratio	Posterior Prob.

**P(action slip)**	**0.5**	**0.145**	**.072**	**[.007,.291]**	**51.2**	0.98*
	3	−0.002	.004	[−.013,.005]	0.40	0.28

**Table 5:** Proportion of participants, remaining after initial filtering, that were excluded by each behavioral exclusion criterion, within each training group (200-trial and 600-trial) and across all subjects. The rows labeled “Lenient exclusion criteria” and “Strict exclusion criteria” display the total exclusion rates when applying the first three criteria, and when additionally applying exclusion criteria for satisficing behaviors, respectively. Results presented in the main text were produced under the strict exclusion criteria (see Table S2 for a summary of results using the lenient criteria).

	% excluded																		

Exclusion Criterion	Exp 1a			Exp 1b			Exp 2a			Exp 2b			Exp 2c			Exp 2d			Exp 3		

	200tr	600tr	All	200tr	600tr	All	200tr	600tr	All	200tr	600tr	All	200tr	600tr	All	200tr	600tr	All	All

Missed trials (C1/C2)	0	3.3	1.4	2.9	3.2	3	0	3.1	1.5	3	1.4	2.3	0	0	0	0.7	2.7	1.4	3.3
TTC failure (C2)	36.6	16.4	28	32.9	12.9	23.5	34.8	25	30	37	23.3	31.2	24.7	12.1	19.3	47.6	31.5	42.2	26.2
Few responses (test)	2.4	8.2	4.9	14.3	9.7	12.1	0	1.6	0.8	7	6.8	6.9	6.5	3.4	5.2	2.8	0	1.8	3.3

Lenient exclusion criteria	36.6	26.2	32.2	35.7	21	28.8	34.8	25.0	30.0	40	28.8	35.3	29.9	15.5	23.7	49.0	32.9	43.6	27.9

Consistent errors (test)	28	6.6	18.9	18.6	6.5	12.9	24.2	12.5	18.5	3	2.7	2.9	20.8	15.5	18.5	20.0	12.3	17.4	4.9
Random spam (test)	31.7	11.5	23.1	10	8.1	9.1				21	15.1	18.5	27.3	20.7	24.4	37.2	15.1	29.8	29.5
Strategic spam (test)	9.8	14.8	11.9	2.9	0	1.5				9	5.5	7.5	2.6	0	1.5	10.3	8.2	9.6	6.6

Strict exclusion criteria	54.9	41	49	37.1	22.6	30.3	39.4	29.7	34.6	50	38.4	45.1	46.8	31.0	40.0	71.7	46.6	63.3	39.3
